# Streamlining pediatric musculoskeletal infection workup: the why, what, and how of rapid MRI protocols

**DOI:** 10.1007/s00247-026-06552-y

**Published:** 2026-03-06

**Authors:** Rohan M. Shah, Ashishkumar K. Parikh, Romie F. Gibly, Jonathan D. Samet

**Affiliations:** 1 Northwestern University Feinberg School of Medicine, 420 East Superior Street, Chicago, IL 60611 United States; 2 Ann & Robert H. Lurie Children’s Hospital of Chicago, Chicago, United States; 3 Emory University School of Medicine, Atlanta, United States

**Keywords:** Rapid MRI, Rapid protocol, Musculoskeletal infection, Osteomyelitis, Abscess

## Abstract

Acute musculoskeletal infection is a common pediatric emergency that requires early diagnosis and management, without which adverse sequelae can include functional impairment, limb deformities, persistent infection, progression to sepsis, and mortality. Magnetic resonance imaging (MRI) is the gold standard for diagnosis. In recent years, there has been growing interest in the use of rapid MRI protocols for acute pediatric musculoskeletal infection, with benefits including quicker scan times with decreased need for sedation and contrast. In the present study, we discuss key considerations, practical challenges, and clinical applications of rapid musculoskeletal MRI in children. We also include various case examples of rapid MRI scans with a practical guide for interpretation to be used by the practicing radiologist. Rapid MRI protocols ultimately present a powerful diagnostic tool that can condense the workup of patients being evaluated for acute musculoskeletal infection, and their incorporation into hospital systems should continue to be investigated.

## Introduction

Acute musculoskeletal infections including osteomyelitis, septic arthritis, and myositis, are common pediatric emergencies that affect an estimated 2 to 20 per 100,000 children annually [1]. Without early diagnosis and treatment, adverse sequelae can include progression to sepsis, functional impairment, joint destruction, limb deformities, chronic infection, and mortality [2]. Radiographs are often the first imaging test, but magnetic resonance imaging (MRI) is the mainstay for diagnosis of acute osteomyelitis and potential complications, including abscess detection [3]. MRI is vital to triage patients into observation/no treatment, versus medical or surgical management. Therefore, there is great value in achieving rapid diagnosis of musculoskeletal infection in children. Rapid MRI protocols are successful in a variety of other pediatric indications including stroke, hydrocephalus, appendicitis, ovarian torsion, and more [4–11]. These protocols are especially relevant given the continued increase in imaging volumes across the USA [12]. Despite this urgency, there is limited use of rapid protocols for musculoskeletal infection nationally, related to a lack of accepted consensus for protocols and other barriers [13].

In this review, we discuss key considerations, practical challenges, and clinical applications of rapid musculoskeletal MRI in children. This article reflects our institutional experiences and current data regarding implementation of rapid protocols in the acute pediatric setting. It is not intended to represent a consensus statement or universally established best practice.

### What are the pros and cons of traditional MRI with contrast and sedation for acute musculoskeletal infection?

Sedation is a common practice in pediatric MRI to produce diagnostic quality images in children who cannot reliably lie still. The goal is to successfully complete the exam without numerous repeated sequences due to motion artifacts. Sedation requires an anesthesia or sedation team which becomes a limiting factor for scheduling exams and is exacerbated by off-hours requests [14]. This leads to delays in scan initiation, requiring patients to stay “nothing by mouth” in anticipation for their upcoming scans and may delay treatment initiation [15]. The diagnosis and management of musculoskeletal infection often hinge on the MRI results, leading to a ripple effect of delays which results in longer hospital stays and cost to the patient [16]. There are reported risks associated with anesthesia in children as well [17, 18]. Shorter scan times can make it easier to tolerate lying still, and child life specialists have been shown to reduce the need for anesthesia and improve efficiency in the MRI department [19]. Despite best efforts, some children will require sedation to complete their MRI. However, unsedated MRI can often be attempted initially, and thus utilize sedated MRI only if it is unsuccessful. Of note, there is currently limited data regarding success rates without sedation, specifically in younger children.

Intravenous contrast is commonly used in MRI exams for MSKI, yet multiple studies have challenged the need for contrast to make accurate diagnoses in this setting [20–23]. Contrast adds multiple sequences and significant time to the MRI exam and requires intravenous access which may be another difficulty for children. Additionally, gadolinium contrast has been shown to accumulate in bodily tissues, yet the long-term risks are unknown [24]. Allergic-like contrast reactions are rare but can be severe [25]. Contrast has been shown to be valuable in certain circumstances to detect osteomyelitis limited to the unossified portions of bone in infants and small children as it can potentially be occult on non-contrast sequences [26]. Given the potential risks and downsides discussed, judicious use of sedation and contrast is critical for efficiency and safety in the workup of these patients. As such, Table 1 can serve as a guide for radiologists and providers when contemplating a rapid MRI.

**Table 1:** Indications and contraindications for the rapid MSK protocol

Indications	Relative contraindications	Contraindications
Acute infection	Chronic infection	Tumors
Over 18 months old	Under 18 months old – due to reported difficulty with unossified epiphyses	Trauma/sports – e.g., fractures, meniscal and labral tears
Serial exams after negative initial exam	Complicated cases	Vascular anomalies

### What is a rapid MRI for acute pediatric musculoskeletal infection?

In recent years, to improve the efficiency of MRI acquisition in children with suspected acute MSKI, rapid sequence protocols without sedation or contrast have been developed [27]. These protocols have been shown to greatly decrease anesthesia rates, scan time, hospital length of stay, and charges without missing actionable diagnoses [27]. In a landmark study, the negative predictive value for the rapid protocol was 97% when the protocol was used correctly [27].

**Fig. 1:**
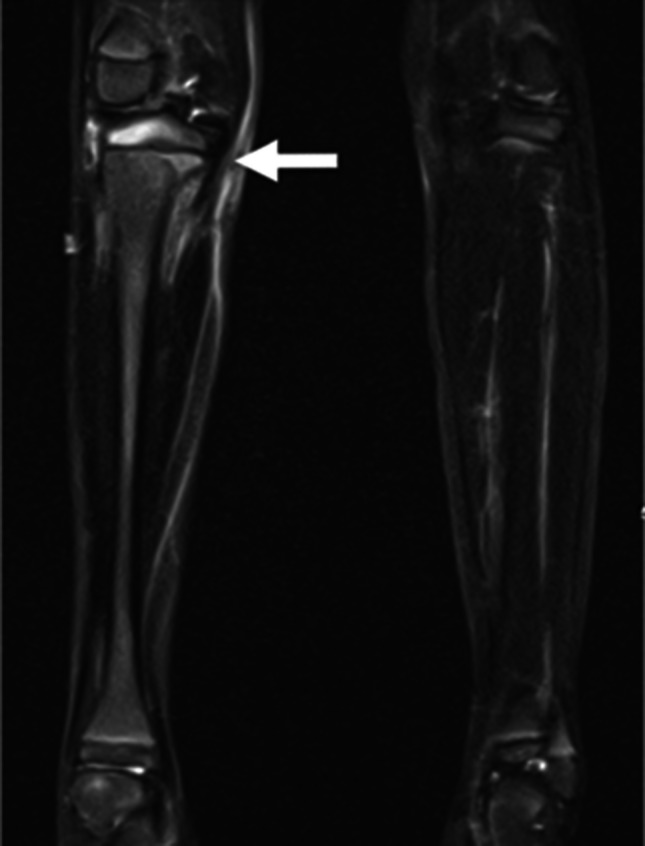
Osteomyelitis in a 6-year-old girl with difficult-to-localize right leg pain and elevated inflammatory markers. Initial large field of view (FOV) coronal STIR image from the rapid MRI protocol helps localize the site of infection to the right proximal tibia (*arrow*). There is bone marrow edema of the right proximal tibial metaphysis and epiphysis with surrounding soft tissue edema consistent with osteomyelitis

Rapid MSKI protocols seek to minimize the time in the MRI scanner by removing sequences that are less essential for diagnosis. Each sequence is optimized for speed and fluid detection. Unlike adult infections, children can present with non-localizing symptoms; and therefore, the rapid protocol must include large field of view (FOV) images to help focus the exam on the pathologic area (Fig. 1). At our institution, for patients with unclear or non-localizing symptoms, the technologist and radiologist can work together after an initial wide FOV sequence to localize the remaining sequences, further decreasing scan area, and thus time. Alternatively, when radiologist support is not feasible, scans could include a broader area, while still preserving the benefits of decreased sedation/contrast.

In the common scenario of the limping child, bilateral lower extremity coronal short tau inversion recovery (STIR) sequences of the thighs and calves are very useful. After the area of concern is chosen, the exam shifts to small FOV sequences. This includes an axial, coronal, and sagittal T2-weighted fat-suppressed sequence, a coronal T1-weighted sequence, and axial diffusion-weighted imaging (DWI) with an accompanying apparent diffusion coefficient (ADC) map. The T2-weighted sequences are essential to detect fluid collections and edema in the bone marrow or soft tissues. The T1-weighted sequence excels at detecting decreased signal in the bone marrow, depicting osteomyelitis. Diffusion-weighted imaging is used to facilitate abscess detection and serves as the alternative to contrast in rapid MSK protocols. At least two *b* values should be used to produce the ADC map, one low and one high value. As in other tissues, restricted diffusion is defined as high signal on the high *b* value with associated low signal on the ADC map. Variations of the described sequences can occur with protocols at different institutions, but should include fluid-sensitive sequences and a non-fat saturation T1-weighted sequence [27] (Table 2). In addition to limiting protocols to essential sequences, newer techniques have emerged that can further reduce scan times. Some of these include acceleration techniques, parallel imaging, radial acquisition, compressed sensing, and simultaneous multi-slice acquisition.

**Table 2:** Example of rapid MRI sequences

Non-localizing clinical impression^a^	Localized or focal clinical impression^a^
Coronal STIR half-Fourier acquisition single-shot turbo spin-echo (large field of view)	Coronal T1-weighted TSE
Coronal T1-weighted turbo spin echo (TSE)	Axial T2-weighted fat-suppressed TSE
Axial T2-weighted fat-suppressed TSE	Coronal T2-weighted fat-suppressed TSE
Coronal T2-weighted fat-suppressed TSE	Sagittal T2-weighted fat-suppressed TSE
Sagittal T2-weighted fat-suppressed TSE	Axial DWI and ADC map
Axial DWI and ADC map	

^a^Of note, per institutional experience from the authors of this review, each individual described sequence should take ~60 s to 90 s on average, depending on the size of the patient and scanner-specific details

**Fig. 2:**
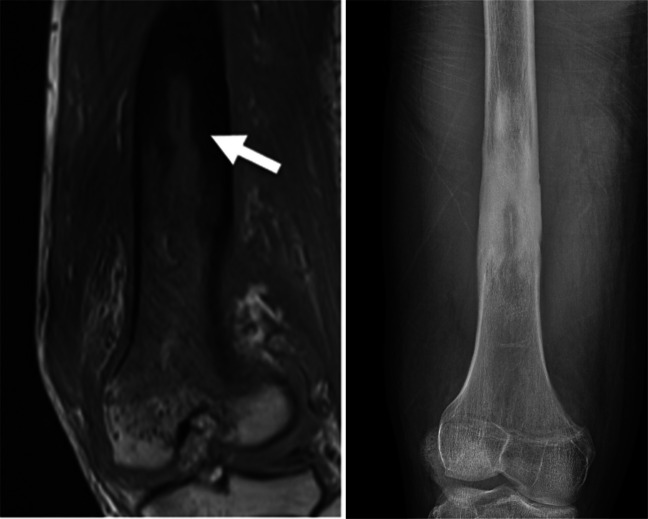
Chronic osteomyelitis in a 16-year-old boy with 5 days of right knee pain, swelling, and elevated inflammatory markers. Though the rapid protocol is intended for acute infection, it is capable of depicting the findings of chronic osteomyelitis. **a** Coronal small FOV T1 image showing extensive T1 hypointensity in the distal femoral metadiaphysis consistent with osteomyelitis. In the distal diaphysis, there is a linear sequestrum (*arrow*) with surrounding hyperostosis and cortical thickening in this patient with unsuspected chronic osteomyelitis. There were also unshown subperiosteal and intraosseous abscesses in this patient. **b** Corresponding radiograph demonstrating a linear bony sequestrum with adjacent medullary canal sclerosis. Ultimately, the patient received both medical management and surgical debridement and reconstruction

Diagnoses that can be made with the rapid MSK protocol include osteomyelitis, subperiosteal abscess, intraosseous abscess, soft tissue abscess, myositis, and septic arthritis. Though the rapid MRI is predominately used in the workup of acute MSKI, unexpected chronic findings of MSKI can also be seen, including bony sequestrum and sclerosis (Fig. 2). The imaging findings associated with these diagnoses will be discussed in detail below.

### Negative exams

Rapid MSKI MRI has been shown to have a high negative predictive value, allowing the emergency department to efficiently discharge patients earlier [27, 28]. The lack of bone marrow signal abnormalities on the T1- and T2-weighted sequences can exclude acute osteomyelitis.

### Acute osteomyelitis

**Fig. 3:**
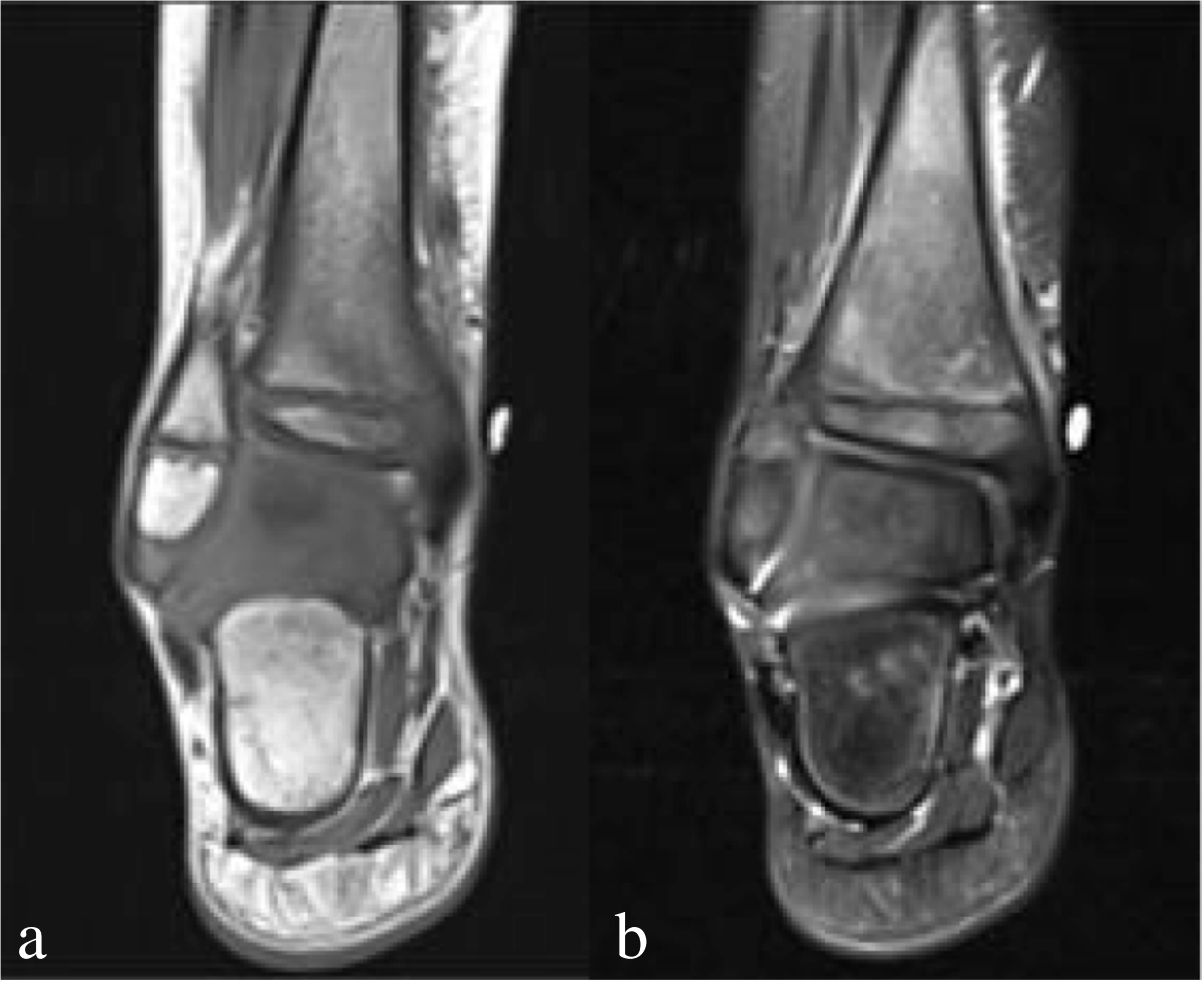
Acute osteomyelitis of the distal tibia in a 5-year-old boy with 5 days of fever, elevated inflammatory markers, and right medial malleolus pain. There are patchy T1 hypointensity (**a**) and corresponding T2 hyperintensity (**b**) in the metadiaphysis. There is surrounding soft tissue edema without abscess formation. The patient ultimately underwent medical management and was discharged

Bone marrow edema is easily detected on T2-weighted sequences which are either a STIR or variation of a T2 fat-suppressed sequence (Fig. 3). As acute osteomyelitis is typically hematogenous, the infection has a predilection for the metaphysis, adjacent to the physis [29]. Conversely, equivocal signal in the diaphysis is less likely to represent osteomyelitis. On T1, the marrow will be low in signal to a varying degree depending on the degree of edema.

### Subperiosteal abscess

**Fig. 4:**
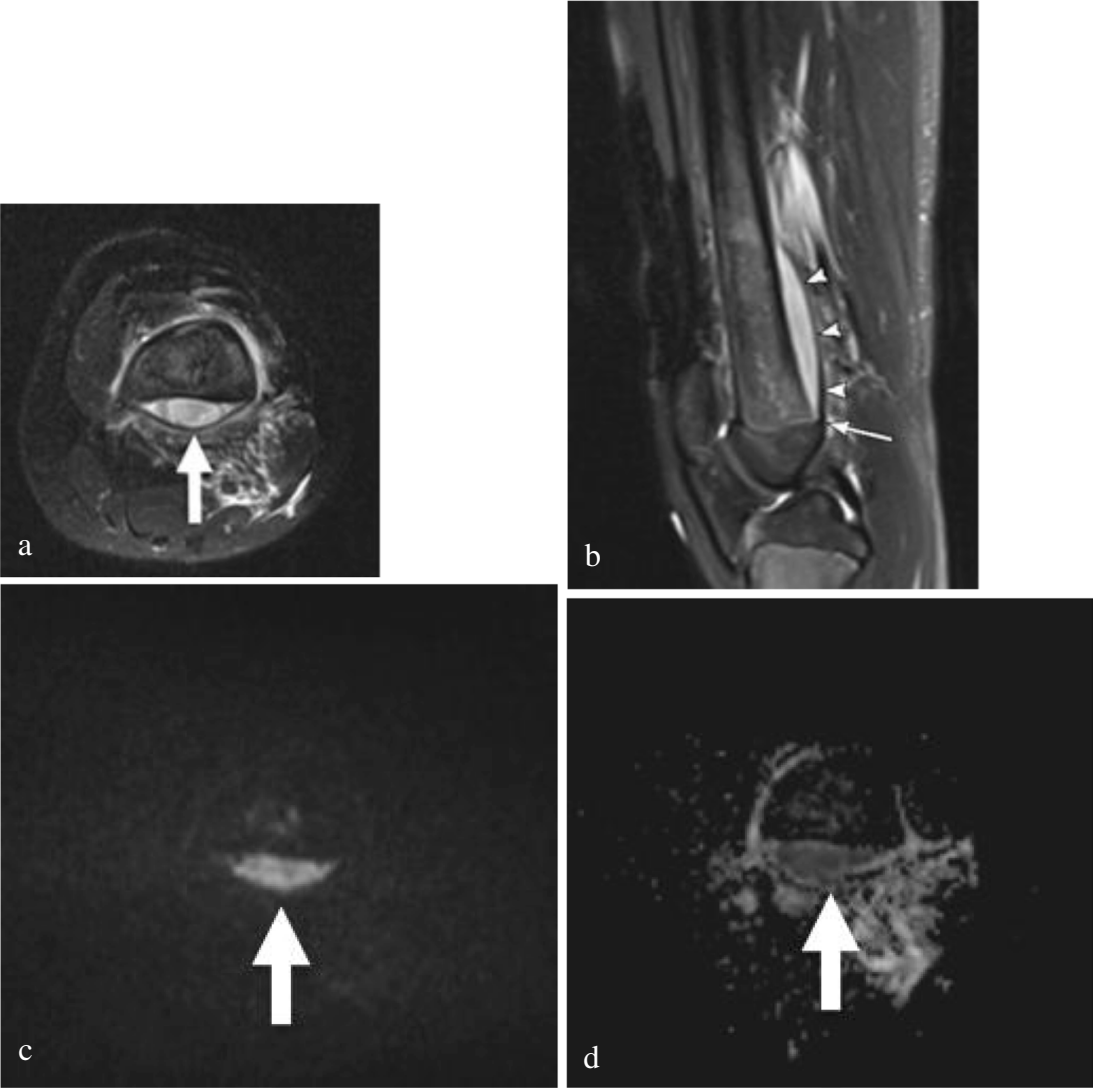
Acute osteomyelitis complicated by subperiosteal abscess in an 11-year-old female patient, with unspecified knee pain. **a** Axial T2 fat-suppressed (FS) image of the distal femur shows a large T2 hyperintense posterior subperiosteal abscess. **b** Sagittal T2 FS image shows the T2 hypointense periosteum lifted by the subperiosteal abscess (*arrowheads*). Note, the abscess tapers and does not extend beyond the level of the perichondrium (*arrow*). Axial DWI b-800 s/mm^2^ (**c**) and ADC (**d**) images show increased signal intensity on the DWI with corresponding low signal on the ADC image, consistent with restrictive diffusion within the subperiosteal abscess (*arrows*). The patient ultimately underwent surgical incision and drainage before discharge

As osteomyelitis progresses, it can extend beyond the cortex into the subperiosteal space [30]. The periosteum is lifted from the cortex due to Sharpey’s fiber laxity in children [31]. The periosteum is hypointense on T2-weighted images and clearly delineated when it is juxtaposed next to the T2 hyperintense subperiosteal fluid collection (Fig. 4). The periosteum can separate from the cortex depending on the size of the abscess but is typically resisted by the perichondrium at the level of the physis. If severe, the collection can break through the periosteum into the surrounding soft tissues. The abscess may demonstrate restricted diffusion on DWI.

### Intraosseous abscess

**Fig. 5:**
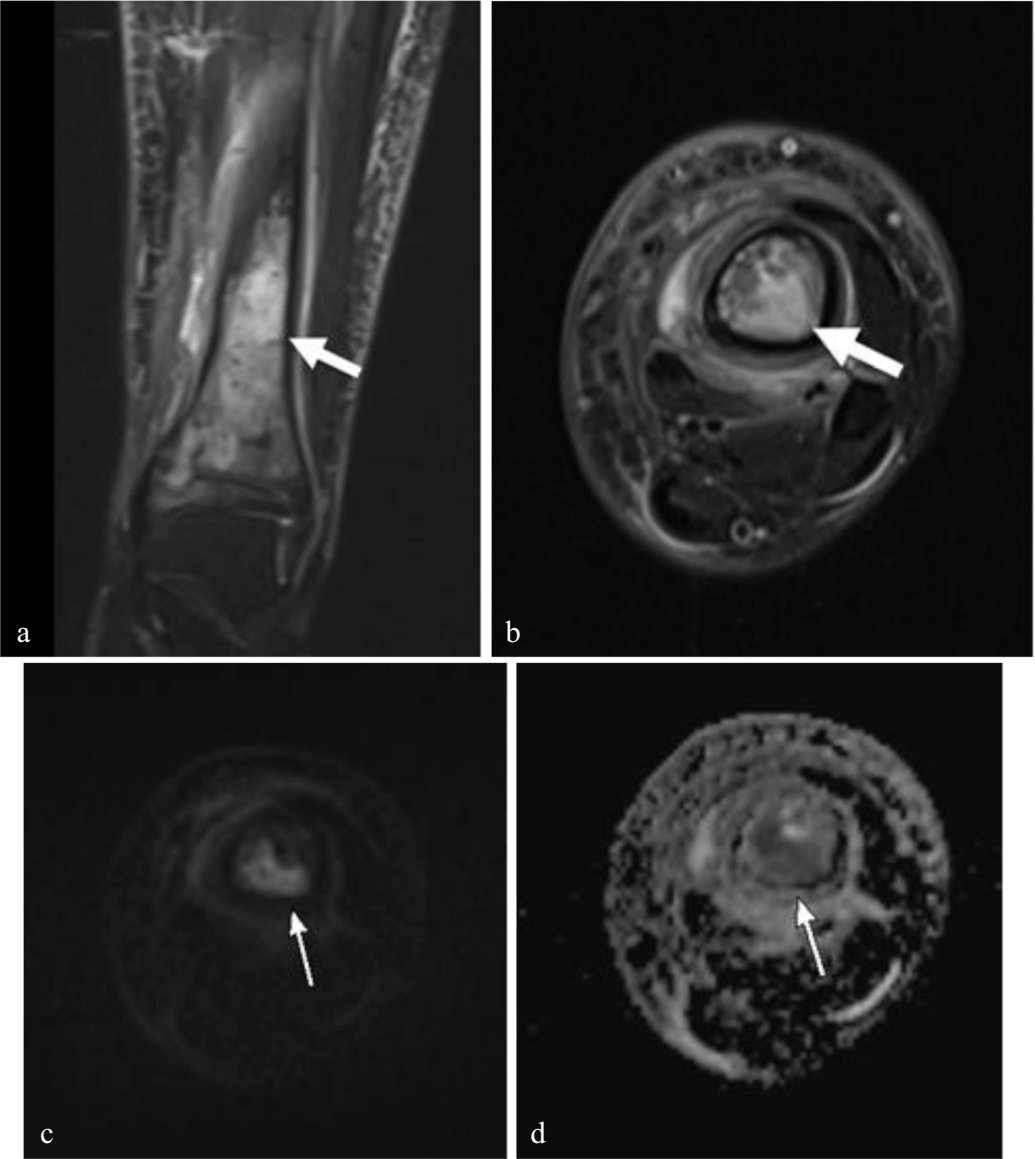
Osteomyelitis complicated by intraosseous abscess formation in a 12-year-old boy with cellulitis of the left lower extremity that did not improve after an 8-day course of antibiotics. Coronal T2 FS (**a**) and axial T2 FS (**b**) images show extensive osteomyelitis of the distal tibia with a focal T2 hyperintense intraosseous abscess (*arrows*). Corresponding high signal on DWI b-800 s/mm^2^ (**c**) and low signal on the ADC map (**d**) are consistent with restricted diffusion of an abscess. The patient ultimately underwent medical management and surgical drainage prior to discharge

In some instances, a phlegmon or abscess can form in the bone. In the subacute setting, this is known as Brodie’s abscess [32]. These are usually found in the same location as acute osteomyelitis, in the metaphysis or metaphyseal equivalents, in periphyseal locations. They will be T2 hyperintense and T1 hypointense centrally. A penumbra sign, or T1 hyperintense peripheral rim, supports the MRI diagnosis of intraosseous abscess (Fig. 5). The abscess may demonstrate restricted diffusion on DWI. It should be noted that intraosseous abscesses often form in subacute cases, and the initial studies on rapid MRI have largely focused on acute osteomyelitis, underscoring the importance of ongoing research.

### Soft tissue abscess

**Fig. 6:**
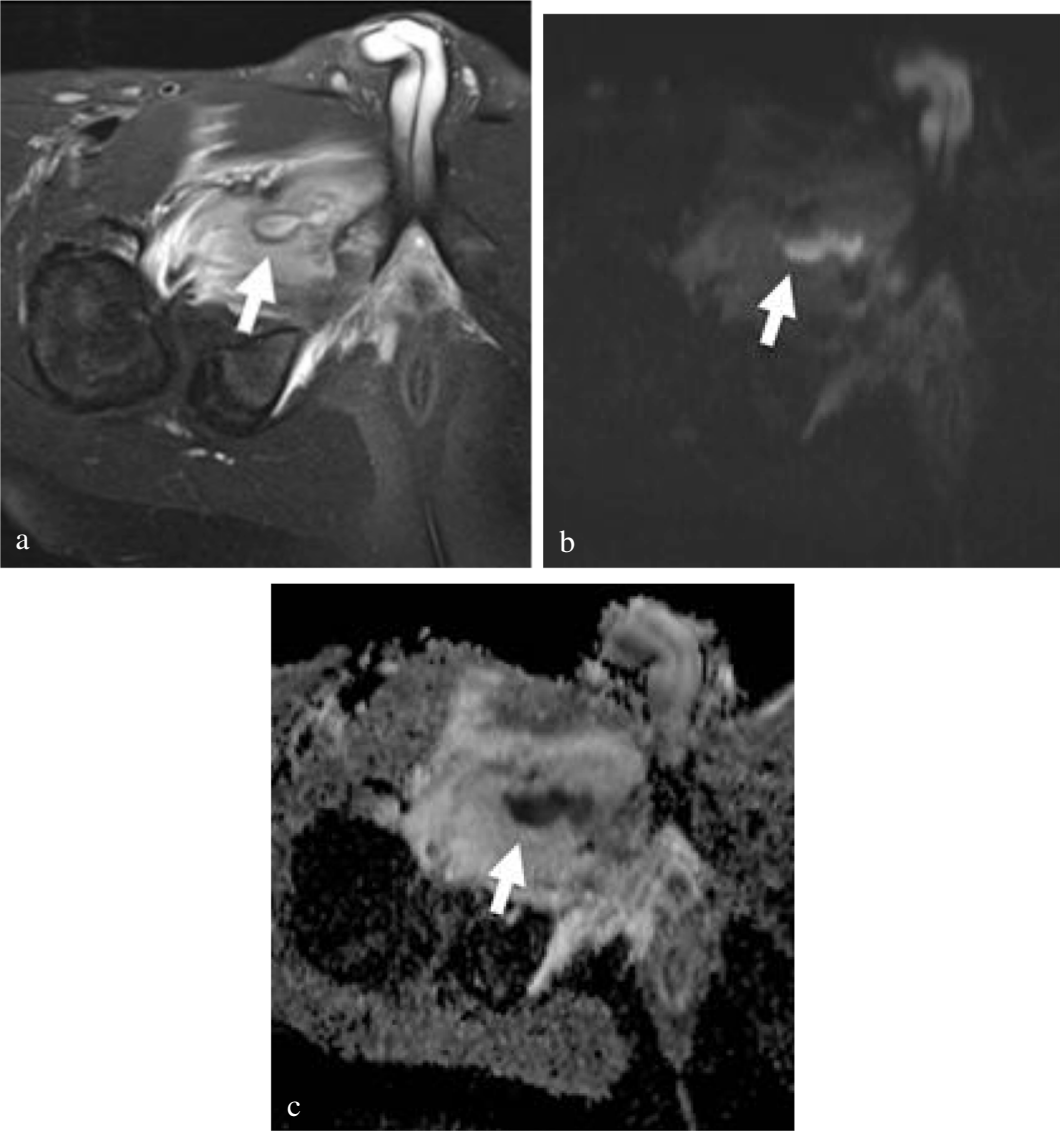
Osteomyelitis of the right ischiopubic synchondrosis and adjacent pyomyositis in a 12-year-old boy with atraumatic right hip pain and fever. **a** Axial T2 FS shows acute myositis in the right adductor musculature with a dumbbell-shaped fluid collection centrally. Corresponding high signal on DWI b-800 s/mm^2^ (**b**) and low signal on the ADC map (**c**) are consistent with restricted diffusion of an abscess. The patient underwent interventional radiology drainage and medical management before discharge

Soft tissue abscesses include intramuscular and subcutaneous abscesses [33]. They are recognized with T2 hyperintense or intermediate signals and are hypointense on T1. They will often demonstrate restricted diffusion on DWI (Fig. 6).

### Septic joint

**Fig. 7:**
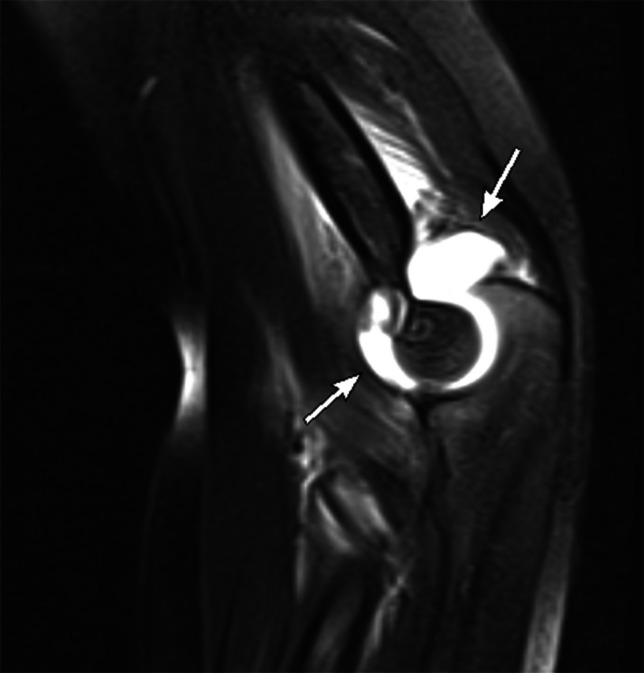
Septic joint in a 6-year-old boy with 4 days of right elbow pain without fevers. Sagittal T2 FS image of the elbow shows a large joint effusion with surrounding soft tissue edema. Though the effusion in isolation is nonspecific, the rapid MRI readily detects pathologic joint effusions. This patient was ultimately managed with operative irrigation and drain placement

Similar to traditional MRI, pathologic joint effusions will demonstrate increased T2 hyperintense fluid in the joint space greater than physiologically expected (Fig. 7). There may be periarticular soft tissue edema and there may be concomitant osteomyelitis that should be investigated. Physical examination, clinical correlation, and procedural sampling of the fluid are key for helping to distinguish septic joints from pathologic joint effusions.

### How to successfully implement a rapid MSKI protocol

The rapid MRI protocol for MSKI should not be viewed simply as a collection of MRI sequences. Implementing it requires strong interdisciplinary collaboration between providers that treat MSKI including radiologists, orthopedic surgeons, and emergency medicine, infectious disease, and hospitalist medicine physicians.

Treatment algorithms or clinical care guidelines can be used to guide appropriate use, limiting the rapid protocol for the setting of suspected acute infection only. Creating an institutional order with the verbiage “infection” can help steer providers away from using this protocol for non-infectious entities such as sports injuries or tumor imaging. Appropriately indicated use is critical since the rapid MRI is optimized for infection and may lack the detail to make subtle non-infectious diagnoses. For subacute to chronic infection or complex cases such as very young patients (<18 months) with unossified bone, postoperative sites, prior osteomyelitis history, retained hardware, or known osteonecrosis such as in Legg-Calve-Perthes or sickle cell disease, traditional MRI with and without contrast may be needed. Since acute MSKI should not be evaluated in an outpatient setting, the rapid protocol can be limited to the ED and inpatient settings, helping avoid misuse. An example of a flowchart algorithm is highlighted in Chan et al. describing one institution’s use of the rapid protocol [27].

Removing contrast and sedation from the workup of MSKI can raise accuracy concerns for some radiologists that are less comfortable interpreting exams in this way. Small group discussions in radiology and with orthopedic colleagues on the risks and benefits of rapid protocols can help mitigate these concerns and fears of missed diagnoses without contrast. A champion for the new protocol in the radiology department and orthopedic department can help drive these discussions and serve as a safety net for clinical questions from colleagues and for quality control of initial cases. Additionally, frequent communication with MRI technologists and MRI physicists can help optimize quality and workflow.

### What if the rapid protocol fails?

Data has shown that the vast majority of patients can successfully complete the rapid MRI exam, with a failure rate due to motion or intolerance of only 9% in a recent analysis [27]. If a patient does fail to complete the rapid exam due to pain or motion, a sedated exam can subsequently be performed without significant downside. Alternatively, a repeat rapid MRI can be attempted with better pain control, or child life support, etc.

### Perspective from the orthopedic surgeon

For the orthopedic surgeon, rapid protocols are extremely appealing as they provide timely answers to understand whether patients will require medical or surgical intervention, or anything at all. By enabling the entire imaging process to be more efficient, rapid protocols make MRI imaging of children easier to obtain, especially when scheduling delays would otherwise hinder access to traditional MRI [34]. Waiting for NPO times, anesthesia, and MRI availability for a traditional sedated contrast scan can introduce significant treatment delays. Earlier acquisition of imaging helps to decrease the risk of missing areas needing intervention which would not be seen on radiographs or ultrasound. A classic example is the sick child with a hip effusion: MRI along with clinical correlation helps the surgeon understand if this represents septic arthritis or a reactive effusion with adjacent osteomyelitis, or both, potentially changing the operative plan [35]. Especially in the setting of septic arthritis, these delays can have real clinical consequences. As seen in Chan et al. most children with MSKI concern present towards the end of the clinical workday [27]. Access to rapid imaging improves the availability of diagnoses before the start of the operating room day, facilitating expeditious treatment if procedural interventions or surgery are required.

One important role the orthopedic surgeon can provide is to reassure the radiologists who are concerned about missing very small abscesses without contrast. The important clinical context is that those small abscesses (such as subcentimeter abscesses in the largest dimension) are rarely a cause for change in management, as these can be extremely difficult to find surgically, and are generally treated medically first [36]. From a treatment perspective, the primary questions that need to be answered by an MSKI MRI are not measured in individual millimeters but relate to identifying the presence and location of an infection, and the presence of any fluid collections or effusions large enough to need drainage.

## Conclusions

Rapid non-sedated non-contrast MRI is a powerful diagnostic tool that can condense the workup and management of patients being evaluated for acute MSKI. Successful implementation requires close collaboration with all providers involved. A small percentage of patients may not tolerate rapid protocols and require traditional sedated imaging, but for the majority of patients, rapid access will improve treatment efficiency, reduce risks, and lower costs.

## Data Availability

No datasets were generated or analysed during the current study.
